# Association Between Stroke and Metabolic Syndrome in a Japanese Population: Jichi Medical School (JMS) Cohort Study

**DOI:** 10.2188/jea.JE20081041

**Published:** 2010-01-05

**Authors:** Yasunori Niwa, Shizukiyo Ishikawa, Tadao Gotoh, Kazunori Kayaba, Yosikazu Nakamura, Eiji Kajii

**Affiliations:** 1 Division of Community and Family Medicine, Center for Community Medicine, Jichi Medical University, Shimotsuke, Tochigi, Japan; 2 School of Health and Social Services, Saitama Prefectural University, Koshigaya, Saitama, Japan; 3 Department of Public Health, Jichi Medical University, Shimotsuke, Tochigi, Japan

**Keywords:** metabolic syndrome X, stroke, cohort studies, incidence, cardiovascular diseases

## Abstract

**Background:**

Metabolic syndrome increases the morbidity and mortality of cardiovascular diseases. However, few studies have examined the association between the incidence of stroke and metabolic syndrome, as defined by Japanese criteria. The aim of this study was to identify the association between stroke and metabolic syndrome, as defined by criteria used in Japan.

**Methods:**

A total of 2205 subjects (920 men and 1285 women) were examined between 1992 and 1995 as part of the Jichi Medical School Cohort Study. Metabolic syndrome was defined using the Japanese criteria. Medical records, computed tomography, and magnetic resonance imaging were used to diagnose stroke. The Cox proportional-hazards model was used to analyze the association between metabolic syndrome and incident stroke.

**Results:**

The prevalence of metabolic syndrome at baseline was 9.0% in men and 1.7% in women. There were 96 incident strokes during an 11.2-year follow-up period, 14 of which occurred in subjects with metabolic syndrome. Among subjects with metabolic syndrome, the age-adjusted hazard ratio (95% confidence interval) for stroke was 1.93 (0.94–3.96) in men and 6.85 (2.68–17.47) in women. After adjusting for age, smoking status, and alcohol drinking status, the hazard ratio was 1.89 (0.88–4.08) in men and 7.24 (2.82–18.58) in women. Age-adjusted hazard ratios associated with having 2 or more components of metabolic syndrome, with and without central obesity, were 2.93 (1.21–7.08) and 3.20 (1.23–8.31) in men and 1.75 (0.69–4.44) and 8.64 (2.82–28.03) in women, respectively.

**Conclusions:**

The presence of metabolic syndrome, as defined by Japanese criteria, increases the risk of stroke; this effect was highly significant among women.

## INTRODUCTION

Metabolic syndrome is defined as a cluster of risk factors—central obesity, hypertension, hyperlipidemia, and impaired glucose tolerance—that increases cardiovascular disease morbidity and mortality.^1^^,^^2^ The third revision of the US Adult Treatment Panel guidelines for cholesterol testing and management was published by the National Cholesterol Education Program in 2001.^3^ In 2005, the Examination Committee of Criteria for Metabolic Syndrome in Japan proposed a new set of criteria for the diagnosis of metabolic syndrome.^4^ In the same year, the International Diabetes Federation presented a new criterion that became an essential component—race- and ethnic-specific measurement of waist circumference.^5^ This parameter was modified to establish a Japanese set point for waist circumference in 2007.^6^ In addition, the American Heart Association/National Heart, Lung, and Blood Institute modified the National Cholesterol Education Program criteria.^7^

In Japan, Health Checkups and Healthcare Advice with a Particular Focus on the Metabolic Syndrome were first implemented in April 2008.^8^ The aims of this program are to prevent middle-aged men and women from developing chronic diseases, thereby reducing medical costs for individuals and the health care system. There is an urgent need for evidence from studies of the Japanese general population regarding the effects of metabolic syndrome, as defined by the Japanese criteria.

Several studies have used various criteria to investigate the prevalence of metabolic syndrome in the Japanese general population; however, few have examined the association between metabolic syndrome, as defined by Japanese criteria, and stroke incidence.^9^^–^^11^ In a previous report, our study group observed no significant association between all-cause mortality and metabolic syndrome, defined by Japanese criteria.^12^

The purpose of the present study was to examine the association between incident stroke and metabolic syndrome, as defined by Japanese criteria, among the Japanese general population.

## METHODS

The Jichi Medical School (JMS) Cohort Study is a prospective population-based study that aims to clarify the risk factors of cardiovascular disease in a Japanese rural population. Details on the JMS Cohort Study design and some descriptive data were published previously.^13^^,^^14^ Baseline data were collected between 1992 and 1995 in 12 rural communities. A total of 12 490 subjects (4911 men and 7579 women) participated in the 12 districts and the waist circumferences of 2286 subjects in 3 of these districts (Takasu, Wara, and Sakuma) were measured. From these 2286 subjects, we excluded 40 subjects who had a previous history of stroke or coronary heart disease, 40 from whom a blood sample could not be obtained, and 1 who was lost to follow-up. Ultimately, 2205 subjects (920 men and 1285 women) were available for observation in the present study. The participation rate for people invited to the mass screening examination was 56%.^15^

Mass screening examinations for cardiovascular disease have been conducted in Japan since 1983, in accordance with the Health and Medical Service Law for the Aged, and the same system was used to collect the present data. In each community, the local government office mailed a personal invitation to all subjects who were enrolled in this study. Trained interviewers used a standardized questionnaire to obtain information about their medical history and lifestyle. Smoking status was classified as current smoker, ex-smoker, or never-smoker; while alcohol drinking was classified as current drinker, ex-drinker, or never-drinker.

Body mass index was calculated as weight (kg) divided by the square of body height (m). Waist circumference was measured at the highest point of the iliac crest. Systolic and diastolic blood pressures were measured with a fully automated sphygmomanometer, the BP203RV-II (Nippon Colin, Komaki, Japan). All blood samples were collected after fasting for at least 8 hours. Serum total cholesterol and triglyceride levels were measured by an enzymatic method (Wako, Osaka, Japan; interassay coefficient of variation (CV): 1.5% for total cholesterol, and 1.7% for triglyceride). High-density lipoprotein cholesterol was measured by phosphotungstate precipitation (using an instrument by Wako, Osaka, Japan; interassay CV: 1.9%). Fasting plasma glucose was measured enzymatically (Kanto Chemistry, Tokyo, Japan; interassay CV: 1.9%).

The subjects who were enrolled in this study were followed-up and their cardiovascular events were investigated and recorded. If they were hospitalized for any reason, their medical records, including duplicate computed tomography scans and magnetic resonance imaging, were checked for evidence of stroke. Each municipal government annually obtained information about subjects who had relocated out of the area. Death certificates were collected from public health centers until the end of 2005, with official permission from the Agency of General Affairs and the Ministry of Health, Labour and Welfare.

The criteria for stroke were sudden onset of a focal and nonconvulsive neurological deficit that persisted for longer than 24 hours; stroke subtype was determined according to the criteria of the National Institute of Neurological Disorders and Stroke.^16^ In this study, cerebral infarction and cerebral hemorrhage were defined as stroke, but cases of subarachnoid hemorrhage were not. All probable cases of stroke in this study were evaluated independently by a diagnosis committee composed of a radiologist and a neurologist, with the aid of computed tomography and magnetic resonance imaging.^13^

Written informed consent for participation in the study was obtained from each responder at the mass screening health checkup. We explained that data would be gathered by using the questionnaire and blood samples, that participants’ health status would be checked, and that their hospital medical records would be examined if a stroke was suspected. All responders agreed to join the study. The Institutional Review Board of Jichi Medical School for Ethical Issues approved this study.

### Metabolic syndrome

The original diagnostic definition of metabolic syndrome in Japan was promulgated by the Examination Committee of Criteria for Metabolic Syndrome in April 2005.^4^ For the purposes of this study, metabolic syndrome was defined as a waist circumference of at least 85 cm in men or 90 cm in women, plus at least 2 of the following: (1) triglycerides ≥1.7 mmol/L (150 mg/dL) or high-density lipoprotein cholesterol <1.0 mmol/L (40 mg/dL), (2) systolic blood pressure ≥130 mm Hg or diastolic blood pressure ≥85 mm Hg, and (3) fasting plasma glucose ≥6.1 mmol/L (110 mg/dL). Subjects who were being treated for diabetes and hypertension were identified by questionnaire at baseline and were included in the study; however, treatment for hyperlipidemia was not taken into account because the questionnaire was not equipped to differentiate those treated for elevated total cholesterol, triglyceride, and lower high-density lipoprotein cholesterol.

### Statistical analysis

All statistical analyses were performed on a personal computer with the Statistical Package for Social Science^®^ (SPSS) for Windows (SPSS Japan Inc., version 11.5, Tokyo, Japan). The results are expressed as the mean ± standard deviation (SD). *P* values were calculated using the *t*-test for variables. Smoking status, alcohol-drinking status, and histories of hypertension and diabetes mellitus were tested using the chi-square test.

The Cox proportional-hazards model was used to calculate the hazard ratios (HRs) for stroke incidence after adjustment for age, smoking status, and alcohol-drinking status, with or without metabolic syndrome, using the Japanese criteria. The crude stroke incidence was calculated per 1000 person-years. A *P* value <0.05 was considered significant.

## RESULTS

The total number of person-years of observation was 24 653, the mean follow-up period (± SD) was 11.2 ± 2.4 years, and the mean age at baseline ± SD was 56.2 ± 12.2 (56.5 ± 12.4 in men and 56.0 ± 12.1 in women). There were 96 incident strokes during the observation period: 54 (5.9%) in men and 42 (3.3%) in women.

Table 1 shows the characteristics of subjects with and without metabolic syndrome (stratified by sex). At baseline, the prevalence of metabolic syndrome, as per the Japanese definition, was 9.0% in men and 1.7% in women. There were no significant differences in smoking or alcohol drinking status between the subjects with and without metabolic syndrome in either sex. The women with metabolic syndrome were older than the women without metabolic syndrome; however, among men, there was no such age difference. With the exception of high-density lipoprotein cholesterol, the values for other parameters were significantly higher in subjects with, as compared to without, metabolic syndrome.

**Table 1.** Clinical characteristics of subjects with and without metabolic syndrome

	Without metabolic syndrome	With metabolic syndrome	*P*-value^a^
Males			
*n* (%)	837 (91.0)	83 (9.0)	
Age (year)	56.3 ± 12.4	57.9 ± 12.1	N.S.
BMI (kg/m^2^)	22.4 ± 2.6	26.4 ± 2.1	<0.001
Waist circumference (cm)	77.9 ± 7.8	90.2 ± 4.6	<0.001
Systolic blood pressure (mm Hg)	127.4 ± 21.2	143.1 ± 17.9	<0.001
Diastolic blood pressure (mm Hg)	76.8 ± 12.2	86.1 ± 11.2	<0.001
Fasting plasma glucose (mmol/L)	5.3 ± 0.9	6.0 ± 1.5	<0.001
Total cholesterol (mmol/L)	4.8 ± 0.9	5.0 ± 0.8	0.01
HDL cholesterol (mmol/L)	1.3 ± 0.4	1.0 ± 0.2	<0.001
Triglyceride (mmol/L)	1.3 ± 1.0	2.2 ± 1.2	<0.001

Current smoking, *n* (%)	404 (49.4)	36 (43.9)	N.S.
Current alcohol drinking, *n* (%)	636 (77.8)	61 (74.4)	N.S.
Diabetes mellitus, *n* (%)	66 (7.9)	32 (38.6)	<0.001
Hypertension, *n* (%)	352 (42.1)	76 (91.6)	<0.001

Females			
*n* (%)	1263 (98.3)	22 (1.7)	
Age (year)	55.9 ± 12.1	62.0 ± 10.7	0.01
BMI (kg/m^2^)	22.9 ± 3.0	28.9 ± 4.5	<0.001
Waist circumference (cm)	73.6 ± 8.7	93.4 ± 3.7	<0.001
Systolic blood pressure (mm Hg)	130.8 ± 22.5	151.0 ± 19.5	<0.001
Diastolic blood pressure (mm Hg)	76.9 ± 13.1	86.4 ± 9.5	<0.001
Fasting plasma glucose (mmol/L)	5.1 ± 0.9	6.3 ± 2.0	<0.01
Total cholesterol (mmol/L)	5.1 ± 0.9	5.5 ± 0.8	0.02
HDL cholesterol (mmol/L)	1.3 ± 0.3	1.1 ± 0.2	<0.001
Triglyceride (mmol/L)	1.1 ± 0.6	2.0 ± 0.9	<0.001

Current smoking, *n* (%)	56 (4.5)	1 (4.5)	N.S.
Current alcohol drinking, *n* (%)	411 (33.1)	7 (31.8)	N.S.
Diabetes mellitus, *n* (%)	80 (6.3)	8 (36.4)	<0.001
Hypertension, *n* (%)	613 (48.5)	21 (95.5)	<0.001

BMI: body mass index.

HDL cholesterol: high-density lipoprotein cholesterol.

N.S.: not significant.

^a^*P*-values were calculated using the *t*-test for variables and the chi-square test for rates.

Data are expressed as the mean ± standard deviation (SD) for variables and as a percentage for rates.

Table 2 shows the crude stroke incidence rate and HRs for metabolic syndrome, calculated by the Cox proportional-hazards model, with the absence of metabolic syndrome as reference. There were 96 incident strokes (in 54 men and 42 women) during the follow-up period. The age-adjusted HRs (95% confidence intervals [CI]) were 1.93 (0.94–3.96) in men and 6.85 (2.68–17.47) in women. After further adjustment for current smoking and alcohol drinking statuses, the HRs were 1.89 (0.88–4.08) for men and 7.24 (2.82–18.58) for women.

**Table 2.** Adjusted hazard ratios in men and women with and without metabolic syndrome

	Males		Females	
		
	Without metabolic syndrome	With metabolic syndrome	Without metabolic syndrome	With metabolic syndrome
All subjects, *n*	920		1285	
Subjects with metabolic syndrome, *n* (%)	837 (91.0)	83 (9.0)	1263 (98.3)	22 (1.7)

Stroke incidence, *n*	45	9	37	5
Crude incidence rate^a^	4.9	10.3	2.6	22.0
HR - model 1^b^ (95% CI)	reference	1.93 (0.94–3.96)	reference	6.85 (2.68–17.47)
HR - model 2^c^ (95% CI)	reference	1.89 (0.88–4.08)	reference	7.24 (2.82–18.58)

Metabolic syndrome was defined using Japanese criteria.

HR: hazard ratio.

CI: confidence interval.

^a^per 1000 person-years.

^b^Hazard ratio adjusted for age.

^c^Hazard ratio adjusted for age, smoking status, and alcohol drinking status.

Next, we classified all subjects into 6 groups by the presence of 0, 1, and 2 or more supplementary components of metabolic syndrome, in men and women with and without central obesity (Tables 3 and 4). Table 3 shows the crude stroke incidence rates and HRs calculated by using the Cox proportional-hazards model. Subjects are classified by the number of supplementary components of metabolic syndrome, the presence of central obesity, and by sex. After adjustment for age and further adjustment for current smoking and alcoholic statuses, the HRs increased in both men and women with 2 or more supplementary components of metabolic syndrome, regardless of central obesity; however, in women, HRs markedly increased in those with central obesity and 2 or more supplementary components of metabolic syndrome, as compared to women without central obesity. There were no strokes among men with central obesity but no supplementary components of metabolic syndrome. There were also no strokes among women with central obesity and fewer than 2 supplementary components of metabolic syndrome.

**Table 3.** Hazard ratios for stroke, by number of supplementary components of metabolic syndrome, presence of central obesity, and sex

No. of supplementary components	*n*	No. of strokes	Crude incidence rate^a^	Model 1^b^		Model 2^c^	
	
				HR	(95% CI)	HR	(95% CI)
Males							
Central obesity (−)	685						
0	259	8	2.8	1.00	reference	1.00	reference
1	298	20	6.2	1.81	(0.79–4.14)	1.73	(0.75–3.97)
≥2	128	13	9.8	2.93	(1.21–7.08)	2.53	(1.02–6.24)
Central obesity (+)	235						
0	36	0	0	—	—	—	—
1	116	4	3.0	1.24	(0.37–4.12)	0.91	(0.24–3.42)
≥2	83	9	10.3	3.20	(1.23–8.31)	2.83	(1.05–7.59)

Females							
Central obesity (−)	1213						
0	510	8	1.4	1.00	reference	1.00	reference
1	503	19	3.3	1.31	(0.57–3.01)	1.31	(0.59–3.10)
≥2	200	10	4.4	1.75	(0.69–4.44)	1.83	(0.72–4.68)
Central obesity (+)	72						
0	14	0	0	—	—	—	—
1	36	0	0	—	—	—	—
≥2	22	5	22.0	8.64	(2.82–26.51)	9.09	(2.95–28.03)

HR: hazard ratio.

CI: confidence interval.

Central obesity: waist circumference ≥85 cm in males or ≥90 cm in females.

^a^per 1000 person-years.

^b^Hazard ratio adjusted for age.

^c^Hazard ratio adjusted for age, smoking status, and alcohol drinking status.

**Table 4.** Hazard ratios of stroke incidence with metabolic components with or without central obesity (using various cut-off value of waist circumference) by sex

No. of supplementary components	WC ≥80 cm					WC ≥85 cm					WC ≥90 cm				
		
	No. of strokes/total	Model 1^a^		Model 2^b^		No. of strokes/total	Model 1^a^		Model 2^b^		No. of strokes/total	Model 1^a^		Model 2^b^	
					
		HR	(95% CI)	HR	(95% CI)		HR	(95% CI)	HR	(95% CI)		HR	(95% CI)	HR	(95% CI)
Males															
Central obesity (−)															
0	5/208	1.00	Reference	1.00	Reference	8/259	1.00	Reference	1.00	Reference	8/280	1.00	Reference	1.00	Reference
1	17/215	2.58	(0.94–7.04)	2.52	(0.92–6.91)	20/298	1.81	(0.79–4.14)	1.73	(0.75–3.97)	23/374	1.83	(0.81–4.10)	1.75	(0.78–3.95)
≥2	9/80	3.76	(1.26–11.27)	3.24	(1.05–9.98)	13/128	2.93	(1.21–7.08)	2.53	(1.02–6.24)	17/169	2.99	(1.28–6.95)	2.50	(1.05–5.95)
Central obesity (+)															
0	3/87	1.88	(0.45–7.88)	2.08	(0.49–8.73)	0/36	—	—	—	—	0/15	—	—	—	—
1	7/199	1.65	(0.52–5.20)	1.41	(0.43–4.63)	4/116	1.24	(0.37–4.12)	0.91	(0.24–3.24)	1/40	0.98	(0.12–7.85)	—	—
≥2	13/131	4.17	(1.48–11.70)	3.83	(1.34–10.92)	9/83	3.20	(1.23–8.31)	2.83	(1.05–7.59)	5/42	4.10	(1.34–12.53)	4.05	(1.32–12.44)

Females															
Central obesity (−)															
0	8/437	1.00	Reference	1.00	Reference	8/481	1.00	Reference	1.00	Reference	8/510	1.00	Reference	1.00	Reference
1	16/388	1.32	(0.56–3.09)	1.27	(0.54–3.00)	17/455	1.31	(0.56–3.05)	1.26	(0.54–2.94)	19/503	1.35	(0.59–3.10)	1.31	(0.57–3.01)
≥2	8/127	1.83	(0.69–4.89)	1.96	(0.73–5.27)	9/171	1.76	(0.68–4.58)	1.84	(0.71–4.79)	10/200	1.75	(0.69–4.44)	1.83	(0.72–4.64)
Central obesity (+)															
0	0/87	—	—	—	—	0/43	—	—	—	—	0/14	—	—	—	—
1	3/151	0.52	(0.14–1.96)	0.53	(0.14–2.12)	2/82	0.62	(0.13–2.95)	0.70	(0.15–3.34)	0/36	—	—	—	—
≥2	7/95	2.30	(0.88–6.38)	2.42	(0.87–6.72)	6/51	3.83	(1.33–11.07)	4.38	(1.51–12.75)	5/22	8.64	(2.82–26.51)	9.09	(2.95–28.03)

HR: hazard ratio.

CI: confidence interval.

WC: Waist circumference.

^a^Hazard ratios adjusted for age.

^b^Hazard ratio adjusted for age, smoking status, and alcohol drinking status.

Table 4 shows HRs calculated using the Cox proportional-hazards model for stroke incidence with 0, 1, and 2 or more supplementary components of metabolic syndrome, in men and women with and without central obesity, using subjects with no supplementary components of metabolic syndrome as a reference. The HRs for stroke were calculated for both sexes using different cutoffs for waist circumference (80, 85, and 90 cm) and the number of supplementary components of metabolic syndrome, as defined by the Japanese criteria. The HRs for stroke among subjects with 2 or more supplementary components of metabolic syndrome were higher in subjects with, as compared to without, central obesity, when the cutoffs for waist circumferences were 90 cm in men and 85 cm in women.

## DISCUSSION

We investigated the associations between stroke and metabolic syndrome, as defined by Japanese criteria, in the general Japanese population. Our findings suggest that metabolic syndrome was associated with an increased incidence of stroke; this effect was statistically significant in women.

Several studies have examined the association between stroke incidence and metabolic syndrome in Japan.^9^^–^^11^^,^^17^^,^^18^ In the Hisayama Study, metabolic syndrome, as defined by the modified National Cholesterol Education Program Adult Treatment Panel III criteria, was associated with increased morbidity for cardiovascular diseases, including stroke.^18^ In the NIPPON DATA 80, metabolic syndrome, as defined by the modified National Cholesterol Education Program Adult Treatment Panel III using body mass index instead of waist circumference, was associated with higher incidences of ischemic stroke and ischemic heart disease.^17^ However, few such studies have used the Japanese definition of metabolic syndrome.^9^^–^^11^ Saito et al reported that the overall prevalence of metabolic syndrome, as defined by the Japanese criteria, was 6.4%, that the sex- and age-adjusted HR for stroke was 0.82, and that metabolic syndrome was not associated with stroke.^9^ They suggested that the Japanese criteria for metabolic syndrome should include 1 requisite component—waist circumference. The use of such a definition weakens the effects of atherosclerotic risk factors (eg, glucose intolerance, hypertension, and dyslipidemia). Takahashi et al reported that the prevalence of metabolic syndrome, defined by the Japanese criteria, was 11.0% in men and 1.1% in women, and that the HR adjusted for age and smoking status was 23.1 in women. They suggested that metabolic syndrome, as defined by the Japanese criteria, was associated with stroke in women but not in men.^10^ Our findings were consistent with theirs; however, their study had a smaller sample size and wider 95% CIs for the HRs.

In the Suita Study, the frequencies of metabolic syndrome in men and women, based on the Japanese criteria, were 17.7% and 5.0%, and the age-adjusted HRs were 1.21 and 2.09; after further adjustment for current smoking and alcohol drinking status, the HRs were 1.27 and 2.05.^11^ In addition, they noted that metabolic syndrome, defined by Japanese criteria, was associated with cardiovascular disease, myocardial infarction, and all-stroke incidence only in women. The investigators suggested that the number of metabolic components might be more strongly associated with cardiovascular disease incidence than the requisite waist circumference criterion. They observed elevated HRs in both sexes, as was the case in the present study; however, they observed a statistically significant association between metabolic syndrome and stroke only in women.

Table 3 shows that, in men, the HRs for stroke incidence in men with 2 or more supplementary components of metabolic syndrome were similar in men with and without central obesity; however, among women, the HRs in women with 2 or more supplementary components of metabolic syndrome and central obesity had a higher risk of stroke than did those without central obesity. The prevalence of central obesity, which is included in the Japanese diagnostic criteria for metabolic syndrome, was 25.5% in men and 5.6% in women. As compared with other populations, the proportion of women with metabolic syndrome was not low.^12^ However, the 95% CIs of the HRs are likely to be wider for women because of the low prevalence of metabolic syndrome.

Some have reported that waist circumference is positively associated with the risk of cardiovascular events.^19^^,^^20^ In our study, there were no strokes either among men with central obesity and no supplementary components of metabolic syndrome or among women with central obesity and 0 or 1 supplementary component of metabolic syndrome (Table 3). Different waist circumference cutoffs (80, 85, and 90 cm) were used to divide the subjects into 6 groups (Table 4). When the waist circumference cutoff was 90 cm for men and 85 cm for women, the HRs for stroke were higher among subjects with central obesity and 2 or more supplementary components of metabolic syndrome than those for subjects without central obesity. Consequently, we believe that the appropriate cutoffs for waist circumference in the Japanese criteria for metabolic syndrome are 90 cm in men and 85 cm in women; moreover, our findings indicate that, in addition to increased waist circumference, the combination of central obesity and 2 or more supplementary components of metabolic syndrome is associated with a higher risk for stroke.

Waist circumference is a requisite in the diagnostic definitions of both the Japanese and International Diabetes Federations; however, the World Health Organization and National Cholesterol Education Program Adult Treatment Panel III criteria include waist circumference as only one of several components. The Japan Diabetes Complication Study (JDCS) observed that there was an association between metabolic syndrome and stroke when the World Health Organization or National Cholesterol Education Program Adult Treatment Panel III diagnostic criteria were used^21^; however, in the same patient group (ie, those observed in the JDCS), there was no significant association with stroke when the diagnostic criteria advanced by the International Diabetes Federation were used.^22^ Their results show that different diagnostic definitions of metabolic syndrome can lead to substantially different assessments of the risks for cardiovascular events in the same population.

In the present study, using waist circumference cutoffs of 90 cm in men and 80 cm in women, which the International Diabetes Federation recommend for Japanese, we re-examined the association between stroke and metabolic syndrome (Table 4). The HRs for stroke increased in both sexes; however, the results were significant only when men with no supplementary components of metabolic syndrome were used as the reference.

The strengths of this study are: (1) it was a longitudinal population-based study, (2) there was almost complete follow-up of subjects who developed cardiovascular disease (including stroke), (3) the follow-up period was long, and (4) fasting blood samples were collected.

The most notable limitation of this study is its small sample size. Because of this, the 95% CIs for the HRs are relatively wide; however, the study is valuable because few longitudinal studies have investigated metabolic syndrome in the Japanese general population. We believe that a longer period of follow-up will solve the problem of small sample size.

Other limitations include: (1) waist circumference was measured at the highest level of the iliac crest; (2) the subjects resided in only 3 rural districts; and (3) drug therapy for dyslipidemia was not identified on the questionnaire. Measurement of waist circumference was not common at health examinations between 1992 and 1995, when the baseline data for the general population were obtained. Even at present, various methods are used to measure waist circumference. According to the Japanese criteria, it should be measured at the level of the umbilicus while the subject is standing and breathing normally. We measured it using the method that is utilized to obtain the waist-to-hip ratio, which is endorsed by the World Health Organization.^23^ However, the use of this method may have underestimated waist circumference.

In conclusion, metabolic syndrome, as defined by the original Japanese criteria, was positively associated with stroke. Furthermore, in women, there was a statistically significant difference in stroke incidence between women with and without metabolic syndrome. We hope that there will be larger and more comprehensive prospective studies of cardiovascular morbidity and mortality in the Japanese general population.
